# An Indirect Comparison Between Nivolumab + Ipilimumab + Two Cycles of Chemotherapy vs. Pembrolizumab + Chemotherapy as First-Line Treatment for Metastatic Non-Small Cell Lung Cancer

**DOI:** 10.3389/fonc.2021.698199

**Published:** 2021-09-13

**Authors:** Panpan Jiang, Ziyang Mao, Qinyang Wang, Xiaohui Jia, Luying Geng, Hong Xu, Lili Jiang, Chengcheng Yang, Min Jiao, Hui Guo

**Affiliations:** ^1^Department of Medical Oncology, The First Affiliated Hospital of Xi’an Jiaotong University, Xi’an, China; ^2^Key Laboratory of Environment and Genes Related to Diseases, Xi’an Jiaotong University, Ministry of Education of China, Xi’an, China; ^3^Centre for Translational Medicine, The First Affiliated Hospital of Xi’an Jiaotong University, Xi’an, China

**Keywords:** pembrolizumab, nivolumab, ipilimumab, non-small cell lung cancer, efficacy, safety

## Abstract

**Background:**

Nivolumab + ipilimumab + two cycles chemotherapy (N-I + chemo, intensive immunotherapy but chemo-light) and pembrolizumab + chemotherapy (Pem + chemo) were both recommended as first-line treatment for metastatic non-small cell lung carcinoma (NSCLC) patients. We conducted this indirect comparison to compare the efficacy of and safety between these two treatments for providing reference for decision making.

**Methods:**

Relevant databases were searched for eligible trials. A well-accepted adjusted indirect treatment comparison (ITC) approach was selected to pool efficacy results and safety outcomes. Subgroup analyses were stratified according to PD-L1 expression and clinical characteristics.

**Results:**

Four eligible randomized trials (CheckMate9LA, KEYNOTE-021G, KEYNOTE 189, KEYNOTE 407) involving 2017 patients were available to analyze. The ITC results suggested that N-I + chemo is comparable to Pem + chemo in OS (HR 1.03, 95% CI 0.82-1.30) and ORR (RR 0.81, 95% CI 0.62-1.06), but tended to yield inferior PFS (HR 1.28, 95% CI 1.04-1.59) than did Pem + chemo. As for safety profiles, N-I + chemo showed no significant difference relative to Pem + chemo in any grade adverse events: (RR 1.03, 95% CI 0.99-1.10), but demonstrated reduced toxicity in chemo-related adverse events, such as anemia (RR 0.63, 95% CI 0.49-0.81), neutropenia (RR0.51, 95% CI 0.33-0.79), and thrombocytopenia (RR 0.38, 95% CI 0.21-0.69).

**Conclusions:**

N-I + chemo is a promising treatment option for providing comparable OS related to Pem + chemo. However, for never smoker female patients, Pem + chemo is preferable to choose for demonstrating favorable OS benefit than N-I + chemo.

## Introduction

In the last decade, the treatment landscape of lung cancer has been revolutionized to the era of immunotherapy, and the most prominent representatives are immune checkpoint inhibitors (ICIs), including medications targeting programmed death receptor 1 (PD-1), programmed death-ligand 1 (PD-L1), and cytotoxic T-lymphocyte-associated protein 4 (CTLA-4) (1, 2). Advances in the 1L immunotherapy treatment of driver mutation-negative metastasis non-small cell lung cancer is remarkable (3). Multiple phase III clinical trials have verified the superior efficacy and acceptable toxicity of ICIs in this population, alone or with other regimens (4–7). However, only 20% of patients obtain long-term survival benefit from a single agent of ICIs (8). Accumulated evidence indicated that a synergistic effect of different regimens contributes to the prolonged survival outcomes (9, 10). Therefore, combination therapy was explored to improve the efficacy and expand the beneficiaries. Specifically, chemo-immunotherapy combinations demonstrated particularly encouraging survival outcomes, and among multiple regimens, pembrolizumab + chemotherapy (Pem + chemo) seemed to yield a better survival benefit (11, 12).

Most recently, a new combination approach, nivolumab and ipilimumab in combination with only two cycles of chemotherapy (N-I + chemo), was designed to administrate in CheckMate9LA (NCT 03,215,706) (13) and showed significantly prolonged OS compared with chemotherapy alone (HR=0.66,95% CI: 0.55 to 0.80) after 12.7 months of follow-up. Thus, this chemo-light combination was approved for previously untreated metastatic NSCLC regardless of PD-L1 expression by the United States Food and Drug Administration (FDA) regulatory in May 2020 (14). N-I + chemo is considered as a new promising treatment option which is associated with improved efficacy in the combination of distinct immune checkpoint inhibitors by functioning in complementary mechanisms. Besides, N-I + chemo is well tolerable due to the short cycle chemotherapeutic agents.

N-I + chemo and Pem + chemo, representing two different combination strategies, were both recommended as first-line treatment for metastatic NSCLC patients without EGFR/ALK mutation. However, there is no available direct comparisons between these two regimens to provide a reference for decision making. Indirect comparison methods (ITC) is an established approach to compare interventions from different trials and the reliability and validity of results has been confirmed to be highly consistent with direct comparisons (15, 16). Thus, we use this method to investigate the potential efficacy and safety difference among N-I + chemo and Pem + chemo in patients with NSCLC in order to offer robust evidence for clinicians, patients, and policy makers to make choices based on comprehensive considerations. Subgroup analysis stratification according to the status of PD-L1 expression and patients’ characteristics also be conducted to guide clinic individualized treatment.

## Methods

### Study Eligibility

We conducted a systematic search on PubMed, Embase, and the Cochrane Central Register of Controlled Trials databases to identify eligible randomized controlled trials performed before January 2021, comparing the efficacy of first-line N-I + chemo or Pem + chemo for metastasis NSCLC patients. Language was restricted to English. Relevant international conferences, such as American Society of Clinical Oncology (ASCO), European Society for Medical Oncology (ESMO), American Association for Cancer Research for Medical Oncology (AACR), and World Conference on Lung Cancer (WCLC) of recent years were also retrieved to avoid missing data. Keywords and relevant variants including “pembrolizumab,” “nivolumab,” “ipilimumab,” “non-small-cell lung cancer,” and “randomized controlled trial” were used to build a search strategy. Study screening and evaluation were conducted by two investigators independently, with disagreements solved by discussion.

### Data Extraction

Two investigators (P.P.J. and Z.Y.M.) independently examined eligible studies in detail and extracted relevant data. As for conflicts, a superior investigator is involved to adjudicate. The outcomes of this study in which we were most interested included overall survival (OS), progression-free survival (PFS), and objective response rate (ORR). We also extracted the following data: treatment-related adverse events (TRAEs), events leading to discontinuation of treatment, and events leading to death. The hazard ratios (HRs) and their 95% confidence intervals (CIs) were acquired for the analysis of survival (OS and PFS), while the dichotomous data was available for ORR and TRAEs analysis. Subgroup analyses were also conducted in OS and PFS according to different PD-L1 expression, histology, sex, age, smoking status and Eastern Cooperative Oncology Group performance status (ECOG PS).

### Statistical Analysis

Traditional meta-analyses were performed to compare the efficacy and safety of Pem + chemo and chemo. The adjusted indirect comparison of N-I + chemo versus Pem + chemo were achieved through an common intervention (chemotherapy), while there is direct comparison between N-I+chemo vs chemo and Pem+chemo vs chemo. The log HR of the indirect comparison was estimated as the following formula: log HR_AB_ = log HR_AC_–log HR_BC_, and its standard error (SE) for the log HR was SE (log HR_AB_) =√ (SE (log HR_AC_)^2^ + SE (log HR_AB_)^2^). RR was calculated similarly using this manner (17, 18). TATA 12.0 (Stata Corporation, College Station, TX) is available for all statistical analyses in this study. A two-sided P of <.05 is statistically significant.

## Results

### Characteristics of the Eligible Studies

#### Study Selection and Quality Assessment

**Figure 1:**
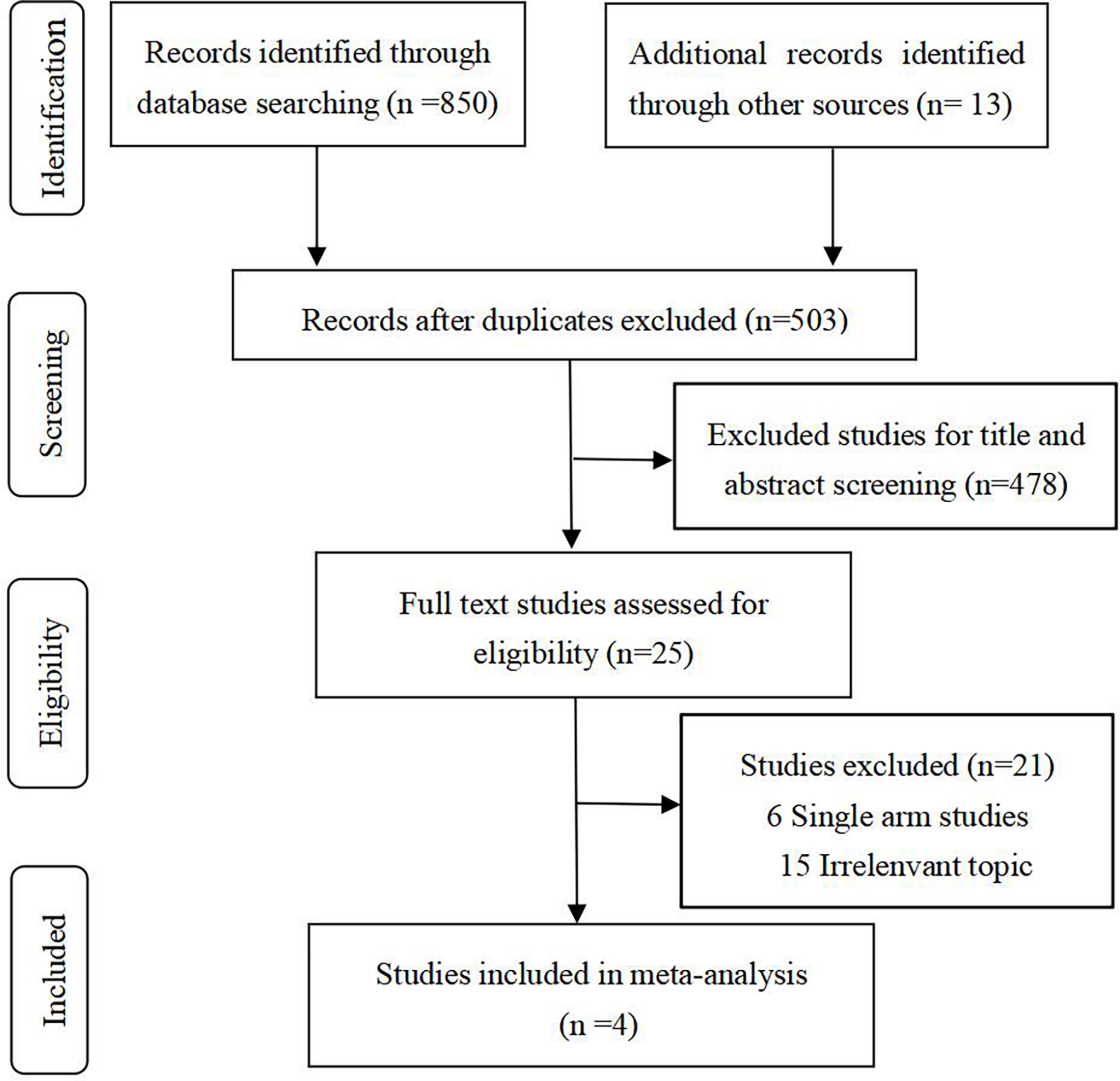
Study flow diagram.

After rigorous selection, 4 relevant RCTs (5, 6, 13, 19) (involving 2017 patients) were identified for inclusion. A specific selection process is illustrated in **Figure 1**.

Updated outcomes were selected for analysis in our study for concluding reliable results. Among 4 included studies, 3 were related to Pem + chemo, and only 1 trial was on N-I + chemo. All studies but KEYNOTE 021G (NCT 02,039,674) were phase 3, international, multicenter trials. Regarding the risk of bias, the Cochrane Collaboration’s risk of bias tool (20) was followed to judge. The main bias was due to insufficient follow-up time and the deficiency of data concerning immune-related adverse events in CheckMate 9LA (NCT 03,215,706). In general, bias assessment results support the high evidence level of our study (**Supplemental Table 1**). Basic characteristics of included studies are presented in **Table 1**. As can be seen from the table, the studies are basically comparable in terms of study design and patient population. Clinical outcomes available for each included RCT are summarized in **Table 2**.

**Table 1:** Baseline characteristics of included trials.

Items	CheckMate 9LA	
KEYNOTE-021G		KEYNOTE-189		KEYNOTE-407	
	(NCT03215706)		(NCT02039674)		(NCT02578680)		(NCT02775435)	
Baseline Characteristics	N-I + chemo	chemo	Pem + chemo	chemo	Pem + chemo	chemo	Pem + chemo	chemo
All eligible patients	361	358	60	63	410	206	278	281
Median age (y)	65.0	65.0	62.5	63.2	65.0	63.5	65.0	65.0
	(35.0-81.0)	(26.0-86.0)	(54.0-70.0)	(58.0-70.0)	(34.0-84.0)	(34.0-84.0)	(29.0-87.0)	(36.0-88.0)
Male sex (%)	70.0	70.0	37.0	41.0	62.0	52.9	79.1	83.6
Region (%)								
East-Asia	NA	NA	NA	NA	NA	NA	19.4	18.5
Non-East Asia	NA	NA	NA	NA	NA	NA	80.6	81.5
ECOG score (%)								
0	31.0	31.0	40.0	46.0	45.4	38.8	26.3	32.0
1	68.0	68.0	58.0	54.0	53.7	60.7	73.7	68.0
2	NA	NA	NA	0.0	0.2	0.0	0.0	0.0
Smoking status (%)								
Cunent/fmmer	87.0	86.0	75.0	86.0	88.3	87.9	92.1	93.2
Never	13.0	14.0	25.0	14.0	11.7	12.1	7.9	6.8
Histologic type (%)								
Squamous	31.0	31.0	0.0	0.0	0.0	0.0	97.5	97.5
Non-squamous	69.0	69.0	100.0	100.0	100.0	100.0	2.2	2.5
Brain metastases, n (%)			15.0	10.0	17.8	17.0	7.2	8.2
Liver metastases, n (%)	19.0	64.0	NA	NA	16.1	23.8	NA	NA
PD-Ll TPS (%)								
≥1	60.0	61.0	65.0	64.0	63.4	62.2	63.3	63.0
1-49	38.0	32.0	32.0	37.0	31.2	28.2	37.1	37.0
≥50	22.0	29.0	33.0	27.0	32.2	34.0	26.3	26.0
<1	40.0	39.0	35.0	37.0	31.0	30.6	34.2	35.2
Follow-up time (mo)	12.7		31.0		46.3		14.3	

N-1, nivolumab plus ipilimumab; Pem, pembrolizumab; chemo, chemotherapy; y, years; NA, not available; ECOG, Eastem Cooperative Oncology Group; PS, perfmmance status; PD-L1 TPS, PD-L1 tumor proportion score; mo, months.

**Table 2:** Infmmation on prima1y outcome of the studies included in meta-analysis.

Source	HR for OS (95%CI)					HR for PFS (95%CI)	ORR(%)		Incidence of TRAEs (%)							
	Overall	PD-Ll ≥ 50%	1%≤PD-Ll <50%	PD-L1 ≥ %	PD-Ll <1%	Overall	EM	CM	Grade 1-5 AEs Grade 3-5 AEsleading to discontinuation leading to death							
									EM	CM	EM	CM	EM	CM	EM	CM
CheckMate 9AL	0.66 (0.55-0.80)	0.66 (0.44-0.99)	0.61 (0.44-0.84)	0.64 (0.50-0.82)	0.62 (0.45-0.85)	0.68 (0.57-0.82)	38.0	25.0	92.0	88.0	49.0	40.0	19.0	7.0	2.0	2.0
Keynote021G	0.56 (0.32-0.95)					0.53 (0.33-0.86)	56.7	30.2	93.0	92.0	41.0	27.0	17.0	13.0	2.0	3.0
Keynote189	0.60 (0.50-0.72)	0.71 (0.50-1.00)	0.66 (0.47-0.93)		0.52 (0.37-0.72)	0.50 (0.41-0.59)	48.3	19.9	99.8	99.0	72.1	67.3	33.6	16.3	7.2	6.9
KEYNOTE-407	0.72(0.58-0.88)	0.79 (0.52-1.21)	0.59 (0.42-0.84)	0.67 (0.51-0.87)	0.79 (0.56-1.11)	0.57 (0.47-0.69)	62.6	38.4	98.2	97.9	69.8	68.2	27.3	13.2	4.3	1.8

OS, overall survival; PFS, progression-fi·ee survival; ORR, objective response rate; TRAEs, treatment-related adverse events; AEs, adverse events; PD-Ll , programmed cell death-ligand 1; 95% Cl, 95% confidence interval (CI); EM, experimental a1m; CM, control aim.

#### Primary and Exploratory Outcomes

**Figure 2:**
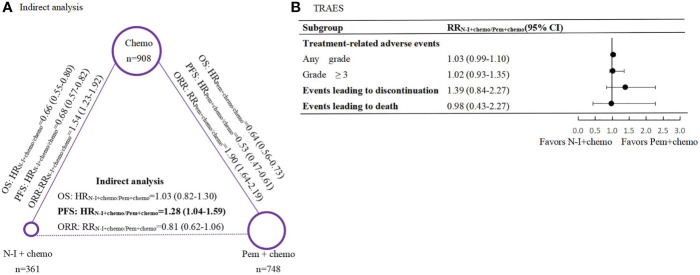
Indirect comparisons of efficacy and safety between N-I + chemo versus Pem + chemo in first-line treatment for patients with advanced NSCLC. **(A)**, Results of indirect analysis for overall survival (OS), progression-free survival (PFS) and objective response rate (ORR) between N-I + chem and Pem + chemo. Each circular represents a treatment. The circle size is associated with the number of enrolled patients. The solid lines represent direct comparisons between the treatments, whereas the dashed line represents the indirect comparison between N-I + chemo versus Pem + chemo. **(B)**, Forest plot of risk ratios (RRs) for treatment-related adverse events (TRAEs) between N-I+chemo and Pem+chemo. N-I, nivolumab plus ipili.mumab; Pem, pembrolizumab; chemo, chemotherapy.

In direct comparison, Pem + chemo appears superior to chemo both in PFS (HR 0.53, 95% CI 0.47-0.61) and OS (HR 0.64, 95% CI 0.56-0.73) (Supplemental Figure 1), while N-I + chemo also showed advantages over chemo in PFS (HR 0.68, 95% CI 0.57-0.82) and OS (HR 0.66, 95% CI 0.55-0.80) (**Figure 2A**). Besides, Pem + chemo demonstrated improved ORR compared with chemo (HR1.90, 95% CI 1.64-2.19). Similar ORR benefit was observed in N-I + chemo vs. chemo (HR 1.54, 95% CI 1.23-1.92). In indirect comparison, N-I + chemo showed no significant difference to Pem + chemo in OS (HR 1.03, 95% CI 0.82-1.30) but is associated with inferior PFS (HR 1.28, 95% CI 1.04-1.59). Regarding ORR, N-I + chemo produced comparable benefits over Pem + chemo (RR 0.81, 95% CI 0.62-1.06) (**Figure 2A**).

### Subgroup Analysis According to PD-L1 Expression

**Figure 3:**
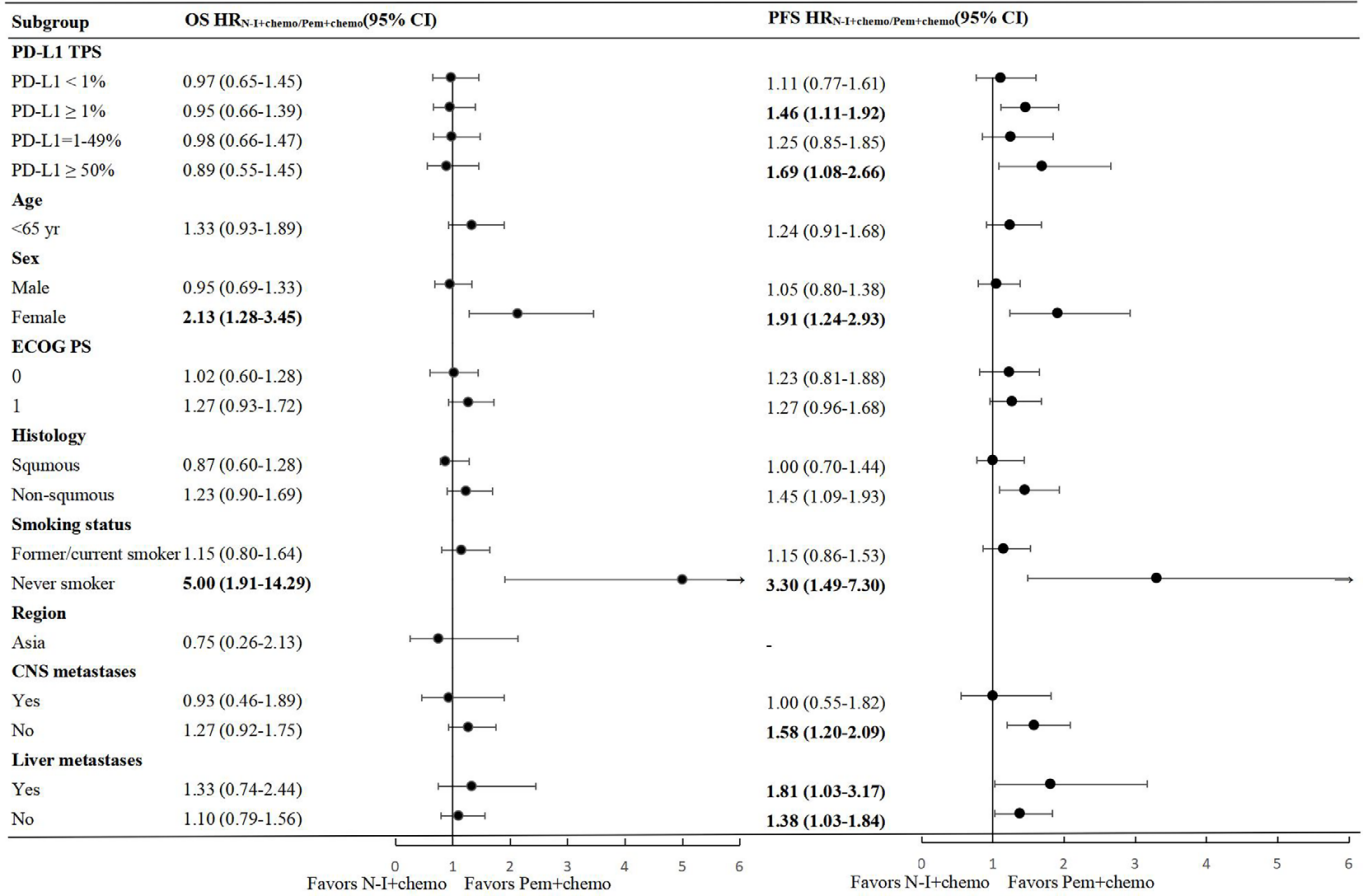
Forest plots of hazard ratios for overall survival and progression free survival in subgroups between N-I + chemo and Pem + chemo. N-I, nivolumab plus ipilimumab; Pem, pembrolizumab; chemo, chemotherapy; CI, confidence interval; ECOG, Eastern Cooperative Oncology Group; PS, performance status.

In the PD-L1 TPS <1% population, direct comparison revealed improved OS and PFS whether N-I + chemo vs. chemo or Pem + chemo v.s chemo (**Supplemental Table 2**). Indirect results indicated that OS (HR 0.97, 95% CI 0.65-1.45) and PFS (HR 1.11, 95% CI 0.77-1.61) were comparable between N-I + chemo and Pem + chemo in this population (**Figure 3**).

In the PD-L1TPS ≥1% population, favorable OS and PFS benefit were observed in both the N-I + chemo and the Pem + chemo groups compared to chemo. Indirect comparison showed N-I + chemo was not inferior to Pem + chemo in OS (HR 0.95, 95% CI 0.66-1.39), but showed inferiority in PFS (HR 1.46, 95% CI 1.11-1.92). Further subgroup analysis was conducted in patients with PD-L1 TPS 1- 49% and 50%. There was no statistical difference between N-I + chemo and Pem + chemo in terms of OS in these two populations, which were generally consistent with results in the overall PD-L1 TPS ≥1% population, indicating the convincing nature of the results. With regard to PFS, N-I + chemo appeared inferior to the PFS benefit compared to Pem + chemo in PD-L1 TPS ≥50% population, with HR of 1.69 (95% CI 1.08-2.66).

### Other Subgroup Analysis

Due to the inconsistency of stratification criteria in subgroup analysis among different trials, patients ≧65 years were unavailable for analysis.

According to indirect comparison, comparable OS and PFS was observed between N-I + chemo and Pem + chemo in pre-stratified subgroups including ECOG PS, CNS metastasis, and liver metastases (**Figure 3**), which is in accordance with the whole population. Significantly, we found females extended the survival time and postponed the tumor progression from Pem + chemo compared to N-I + chemo, with HR 0.81 (95% CI 0.29-0.78) and 0.52 (95% CI 0.34-0.81) for OS and PFS respectively. The same result appears to never smokers, with improved OS (HR 0.20, 95% CI 0.07-0.52) and PFS (HR 0.30, 95% CI 0.14-0.67) benefit from Pem + chemo than N-I + chemo. Besides, non-squamous NSCLC patients showed significant advantage from Pem + chemo than N-I + chemo in PFS (HR 0.69, 95% CI 0.52-0.92) but this advantage was not apparent in OS (HR 0.81, 95% CI 0.59-1.11).

### Safety Analysis

As for safety profiles, our results demonstrated similar risks across multiple safety endpoints between N-I + chemo and Pem + chemo, including any grade AEs (RR1.03, 95% CI 0.99-1.10), grade 3-5 AEs (RR 1.02, 95% CI 0.93-1.35), events leading to drug discontinuation (RR 1.39, 95% CI 0.84-2.27), and events leading to death (RR 0.98, 95% CI 0.43-2.27) (**Figure 2B**). In terms of specific commonly reported TRAEs, N-I + chemo is associated with less hematological toxicity in contrast to Pem + chemo, such as anemia (RR 0.63, 95% CI 0.49-0.81), neutropenia (RR0.51, 95% CI 0.33-0.79), and thrombocytopenia (RR 0.38, 95% CI 0.21-0.69). Nevertheless, the rate of nausea (RR 0.70, 95% CI 0.55-0.90) and colitis (RR 0.38, 95% CI 0.21-0.69) also occurred less frequently in patients who received N-I + chemo (**Table 3**). Direct comparisons of safety between Pem + chemo and chemo are presented in **Supplemental Figure 2**.

**Table 3:** Relative risks for common treatment-related adverse events with N-1 + chemo versus Pem + chemo.

Treatment-related adverse events		RR_N-I+chemo/chemo_ (95%CI)	RR_Pem+chemo/chemo_ (95%CJ)	RR_N-J+chemo/Pem+chemo _(95%CJ)
Rash	Any grade	5.94 (3.19-11.04)	1.68 (1.28-2.19)	3.57 (1.79-7.14)
	Grade ≥ 3	12.67 (0.72-224.13)	1.89 (0.63-5.64)	6.67 (0.31-140.78)
Diarrhea	Any grade	1.76 (1.24-2.50)	1.39 (1.14-1.68)	1.27 (0.85-1.89)
	Grade ≥ 3	7.31 (1.68-31.74)	1.62 (0.87-3.01)	4.54 ( 0.92-20)
Pruritus	Any grade	1.18 (0.87-1.60)	2.08 (1.36-3.18)	0.57 (0.34-0.95)
	Grade ≥ 3	6.82 (0.35-131.63)	0.34 (0.01-8.21)	2.00 (0.03-210.04)
Fatigue	Any grade	1.56 (1.07-2.28)	1.09 (0.94-1.270	1.43 (0.95-2.13)
	Grade ≥ 3	3.90 (0.83-18.23)	1.58 (0.92-2.70)	2.44 (0.48-12.50)
Decreased appetite	Any grade	1.05 (0.75-1.46)	0.95 (0.79-1.13)	1.11 (0.75-1.61)
	Grade ≥ 3	0.97 (0.25-3.87)	1.08 (0.38-3.10)	0.90 (0.16-5)
Asthenia	Any grade	1.18 (0.87-1.60)	0.95 (0.76-1.17)	1.23 (0.85-1.82)
	Grade ≥ 3	0.27 (0.10-1.37)	1.13 (0.62-2.03)	0.33 (0.09-1.22)
Nausea	Any grade	0.75 (0.60-0.93)	1.07 (0.95-1.21)	0.70 (0.55-0.90)
	Grade ≥ 3	1.62 (0.39-6.75)	1.05 (0.52-2.14)	1.54 (0.32-7.69)
Vomiting	Any grade	0.90 (0.63-1.29)	1.30 (1.04-1.63)	0.69 (0.45-1.06)
	Grade ≥ 3	1.17 (0.36-3.80)	0.91 (0.43-1.93)	1.28 (0.32-5.26)
Constipation	Any grade	0.78 (0.50-1.21)	1.13 (0.94-1.36)	0.69 (0.43-1.11)
	Grade ≥ 3		1.08 (0.28-4.11)	
Anemia	Any grade	0.61 (0.49-0.77)	0.97 (0.87-1.09)	0.63 (0.49-0.81)
	Grade ≥ 3	0.40 (0.25-0.65)	0.93 (0.73-1.20)	0.43 (0.25-0.74)
Neutrophil count decreased	Any grade	0.27 (0.63-2.59)	1.38 (0.58-3.26)	0.92 (0.30-2.78)
	Grade ≥ 3	2.20 (0.42-11.59)	1.05 (0.82-1.34)	0.79 (0.39-1.59)
Neutropenia	Any grade	0.58 (0.39-0.86)	1.13 (0.95-1.35)	0.51 (033-0.79)
	Grade ≥ 3	0.71(0.43-1.17)	1.05 (0.82-1.34)	0.68 (0.39-1.19)
Thrombocytopenia	Any grade	0.49 (0.28-0.86)	1.30 (1.04-1.63)	0.38 (0.21-0.69)
	Grade ≥ 3	1.19 (0.50-2.84)	1.22 (0.80-1.85)	0.97 (0.22-4.35)
Colitis	Any grade	10.72 (1.39-82.62)	3.69 (1.33-10.23)	0.38 (0.21-0.69)
	Grade ≥ 3	10.72 (0.60-193.21)	3.11 (0.92-10.51)	3.45 (0.15-100)
Hypothyroidism	Any grade	55.57 (7.74-399.08)	3.88 (1.83-8.25)	14.29 (1.73-117.83)
	Grade ≥ 3	2.92 (0.01-71.55)	3.58 (0.43-29.63)	0.81 (0.02-33.33)
Adrenal insufficency	Any grade	24.37 (1.45-410.08)	1.73 (0.29-10.20)	14.29 (0.50-403.00)
	Grade ≥ 3	8.77 (0.47-162.36)	1.18 (0.18-7.97)	7.69 (0.24-249.20)

N-I, nivolumab plus ipilimumab; Pem, pembrolizumab; chemo, chemotherapy; RR, relative risk.

## Discussion

N-I + chemo and Pem + chemo, representing two different treatment models, were both recommended as first-line treatment options for metastasis NSCLC. It is essential to understand the potential efficacy and safety difference among N-I + chemo and Pem + chemo to provide reference for clinical therapeutic determination. Through comprehensive analysis, our study revealed N-I + chemo (chemo-light) has a comparable OS benefit relative to Pem + chemo, but is associated with a less favorable PFS benefit. Furthermore, any grade AEs as the primary safety endpoint were not observed to be significantly different among N-I + chemo and Pem + chemo. Notably, patients who received N-I + two cycles of chemotherapy experienced less hematologic toxicity.

Current evidence emphasizes the superior survival benefit of Pem + chemo among multiple existing immunotherapies (11, 12, 21) in first-line treatment for metastasis NSCLC. Our results showed N-I + chemo as a chemo-light therapy has comparable OS benefit compared with Pem + chemo, which encourages patients to choose N-I + chemo under similar OS benefit while chemotherapy is intolerable. Considerable OS benefit of this chemo-light combination therapy is attributed to several aspects. First, short cycle chemotherapy can increase tumor immunogenicity by eliminating tumor cells and releasing antigen (10, 22), and is also associated with enhanced PD-L1 expression and potentiated T cell-mediated cytotoxicity while treated with nivolumab (23). Moreover, distinct immune checkpoint inhibitors function in complementary mechanisms with improved efficacy (24, 25). As for insufficient PFS benefit of N-I + chemo compared to Pem + chemo revealed in our study, possible explanation may include inadequate follow-up time and unsatisfactory synergy of nivolumab and ipilimumab. Mature data updated in the future will be discussed further.

Given the expression status of PD-L1 is established biomarkers of the efficacy of immunotherapy (26), subgroup analyses according to different level of PD-L1 were conducted to guide more individualized treatment. However, no significant OS benefit difference was observed across different PD-L1 levels, which is generally identical with the results in the whole population.

Unexpectedly, our study suggests Pem + chemo appears to have significantly superior efficacy in deferring tumor progression compared with N-I + chemo in patients with PD-L1 TPS≥50%. This finding overturns our previous hypothesis that patients with high PD-L1 expression can benefit more from dual immune inhibitors. Similarly, in KEYNOTE-598 (NCT 03,302,234) (27), Pem + ipilimumab failed to improve efficacy compared to pembrolizumab monotherapy in the first-line treatment of metastatic NSCLC patients with PD-L1 > 50%, which also suggests the predictive value of PD-L1 expression is unavailable in dual immune inhibitors. Thus, valuable predictive biomarkers for N-I + chemo need further investigation to identify potential beneficiaries. With regard to the other subgroup analyses, comparable OS and PFS benefits were observed between N-I + chemo and Pem + chemo in most groups, but this result is missing females and never smokers. Intriguingly, Pem + chemo appeared more effective than N-I + chemo in females and never smokers, and there are multiple overlaps in these two populations. This result is in conformity to current research evidence. First, studies have reported that female tumors tend to have less cancer-associated antigens than male tumors (28, 29). This indicates that females have less antigenicity which resulted in a less favorable immunotherapy efficacy in female patients (30). Besides this, the disadvantage in drug pharmacokinetics and pharmacodynamics (31, 32) are both considered to be correlated with a compromised efficacy in females. As for efficacy difference observed in smokers and never smokers, potential explanation may be that smokers have different features of gene mutation (33–35) and functions of immunoregulation (36, 37), which is conductive to the response of immunotherapy. Most recent evidence further indicated smoking can promote PD-L1 expression (38) and increase TMB (8). All these factors lead to the conclusion that smokers derive more from intensive immunotherapy than never smokers. As for superior survival benefit observed in non-squamous NSCLC patients who received Pem + chemo treatment, more clinical data are demanded to confirm our findings.

Besides efficacy, the safety profiles are also an essential concern when administrating regiments. Generally, no significant difference was observed in any grade adverse events among N-I + chemo and Pem + chemo in our study. As we expected, short cycle chemotherapy under the model of N-I + chemo was associated with less hematological toxicity, indicating the application superiority for patients unable to suffer long-term standard chemotherapy. With the recent and continuous application of ICIs, increasing attention has been paid to immune-related toxicities. Noteworthy, combined immune blockage with nivolumab and ipilimumab may increase immune-related adverse events (39, 40). However, due to the sparse data, thorough immune-related adverse events are not available to analyze in our study.

As far as we are aware, our study is the first to explore the difference between N-I + chemo and Pem + chemo in NSCLC to provide valuable insight for informing clinical decision making, although of course, more evidence from real-world and direct comparisons is required to support our findings. Another strength of our study was we performed a comprehensive subgroup analysis to explore the potential efficacy difference in patients with different clinical characteristics. Inevitably, several limitations were encountered in our study. First, head-to-head comparison is lacking and there is methodological limitation of indirect comparison for integrating results of trials with heterogeneity. Besides, the immature OS data of N-I + chemo resulting from insufficient follow- up time might lead to a potential bias. Given these limitations, more reliable results based on mature and individual patient’s data are required. Additionally, the regimens included in our study represent different combination strategies. However, owing to limited trials included, which treatment strategy is preferable to choose in clinical practice has not been answered. With increasing studies attempting dual checkpoint inhibition combination, future studies evaluating these two treatment models are needed to guide study design and treatment selection.

In conclusion, N-I + chemo is a promising treatment option, especially available to patients who are elderly, weak, or unable to suffer through long-term chemotherapy. However, for never smoker female patients, Pem + chemo is preferable to choose for providing superior OS benefit compared to N-I + chemo. Collectively, efficacy and toxicities should be comprehensively taken into consideration and be balanced, for further formulating individualized treatment.

## Data Availability Statement

The original contributions presented in the study are included in the article/**Supplementary Material**. Further inquiries can be directed to the corresponding author.

## Author Contributions

All authors contributed to the article and approved the submitted version. PJ and ZM: Writing - original draft, writing - review and editing. QW and XJ: conceptualization, methodology. LG and HX: investigation, resources, data curation, extracted data from studies, and matched inclusion and exclusion criteria. LJ and CY: software, formal analysis. MJ: visualization, project administration. HG: supervision. All authors had full access to all data, critically revised the paper, approved the final analysis, and took responsibility for all aspects of the work to ensure that issues relating to the accuracy or integrity of any part of the work could be appropriately investigated and resolved.

## Funding

This work was supported by Targeted Therapy Fund of Chinese Society of Clinical Oncology (Y-HR2015-139) and Chinese Society of Clinical Oncology MSD Cancer Research (Y-MSD2020-0247).

## Conflict of Interest

The authors declare that the research was conducted in the absence of any commercial or financial relationships that could be construed as a potential conflict of interest.

## Publisher’s Note

All claims expressed in this article are solely those of the authors and do not necessarily represent those of their affiliated organizations, or those of the publisher, the editors and the reviewers. Any product that may be evaluated in this article, or claim that may be made by its manufacturer, is not guaranteed or endorsed by the publisher.
