# Supplementary material for: An Indirect Comparison Between Nivolumab + Ipilimumab + Two Cycles of Chemotherapy vs. Pembrolizumab + Chemotherapy as First-Line Treatment for Metastatic Non-Small Cell Lung Cancer
Source: Front Oncol. 2021 Sep 13;11:698199. doi: 10.3389/fonc.2021.698199 (PMC8473819; doi:10.3389/fonc.2021.698199)
Supplement: Supplementary file 1 [file DataSheet_1.docx]

|  |  |  |  |  |  |  |
| --- | --- | --- | --- | --- | --- | --- |
| Trial | Sequence generation | Allocation concealment | Blinding | Incomplete outcome data | Selective reporting | Other source of bias |
| KEYNOTE-021G | Adequate | Adequate(Central allocation) | Adequate a | Adequate | Adequate |  |
| KEYNOTE-189 | Adequate | Adequate(Central allocation) | Adequate a | Adequate | Adequate |  |
| KEYNOTE-407 | Adequate | Adequate(Central allocation) | Adequate a | Adequate | Adequate |  |
| CheckMate 9AL | Adequate | Adequate(Central allocation) | Adequate a | Inadequate data b | Adequate | Inadequate data c |
| aThe sponsor, investigator and subject were aware of the treatment administration but the response to treatment was assessed by means of blinded, independent, central radiologic review. b Insufficient follow-up data cAbsence of immune-related adverse events. | | | | | | |

**Supplementary Material**

Abbreviations: N-I, nivolumab plus ipilimumab; Pem, pembrolizumab; chemo, chemotherapy; OS, overall survival; HR, hazard ratio.

TABLE S2. Summary of clinical outcomes according to PD-L1 expression level.

TABLE S1. Quality assessment by Cochrane Collaboration’s tool.

| Subgroup | Pem+chem versus chemo | N-I+chemo versus chemo | N-I+chemo versus Pem+chemo |
| --- | --- | --- | --- |
| PD-L1 < 1% |  |  |  |
| OS HR (95% CI) | 0.64 (0.50-0.81) | 0.62 (0.45-0.85) | 0.97 (0.65-1.45) |
| PFS HR (95% CI) | 0.71 (0.53-0.94) | 0.64 (0.51-0.79) | 1.11 (0.77-1.61) |
| PD-L1 ≥ 1% |  |  |  |
| OS HR (95% CI) | 0.67 (0.51–0.87) | 0.64 (0.50-0.82) | 0.95 (0.66-1.39) |
| PFS HR (95% CI) | 0.67 (0.53-0.84) | 0.46 (0.40-0.54) | 1.46 (1.11-1.92) |
| PD-L1=1-49% |  |  |  |
| OS HR (95% CI) | 0.62 (0.49-0.79) | 0.61 (0.44-0.84) | 0.98 (0.66-1.47) |
| PFS HR (95% CI) | 0.69 (0.51-0.94) | 0.55 (0.43-0.70) | 1.25 (0.85-1.85) |
| PD-L1 ≥ 50% |  |  |  |
| OS HR (95% CI) | 0.74 (0.57-0.97) | 0.66 (0.44-0.99) | 0.89 (0.55-1.45) |
| PFS HR (95% CI) | 0.61 (0.42-0.89) | 0.36 (0.28-0.47) | 1.69 (1.08-2.66) |

FIGURE S1. Direct comparisons of efficacy between Pem + chemo and chemo. Forest plot of hazard ratios (HRs) comparing (A) overall survival, progression-free survival (B) and objective response rate (C) between Pem + chemo

and chemo.


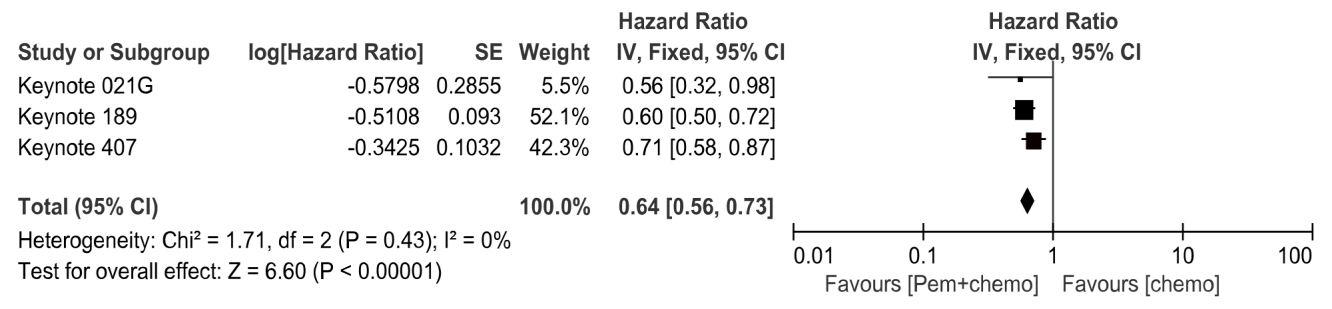


A


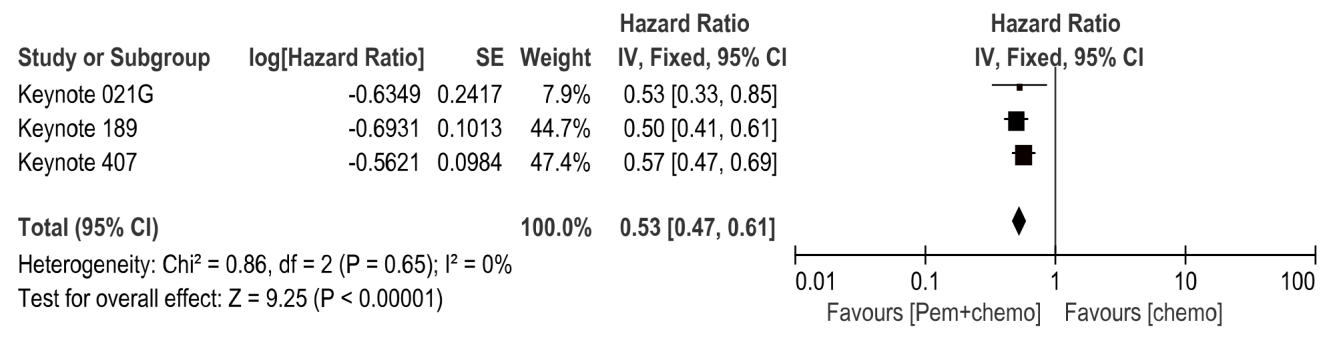


B


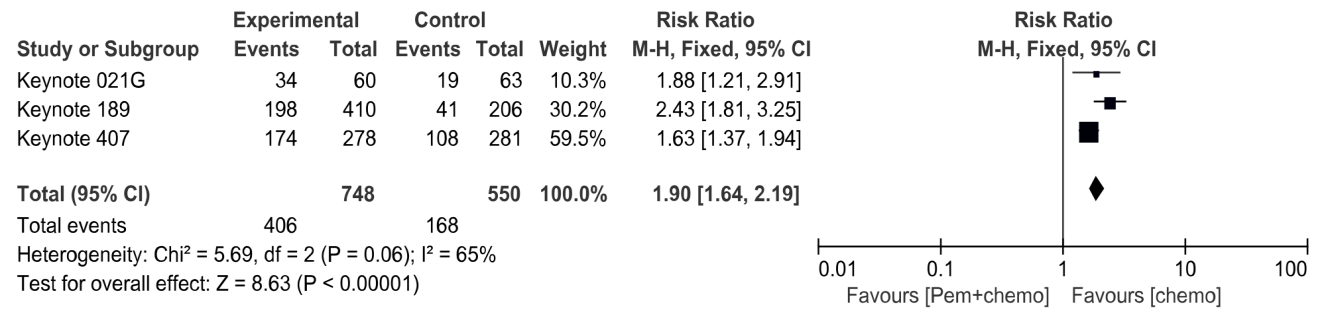


C

FIGURE S2. Direct comparisons of safety between Pem + chemo and chemo. A-D, Forest plot of risk ratios (RRs) for treatment-related adverse events (AEs) between Pem + chemo and chemo. (A) Any grade AEs (B) Grade 3-5 AEs (C) Events leading to discontinuation (D) Events leading to death.


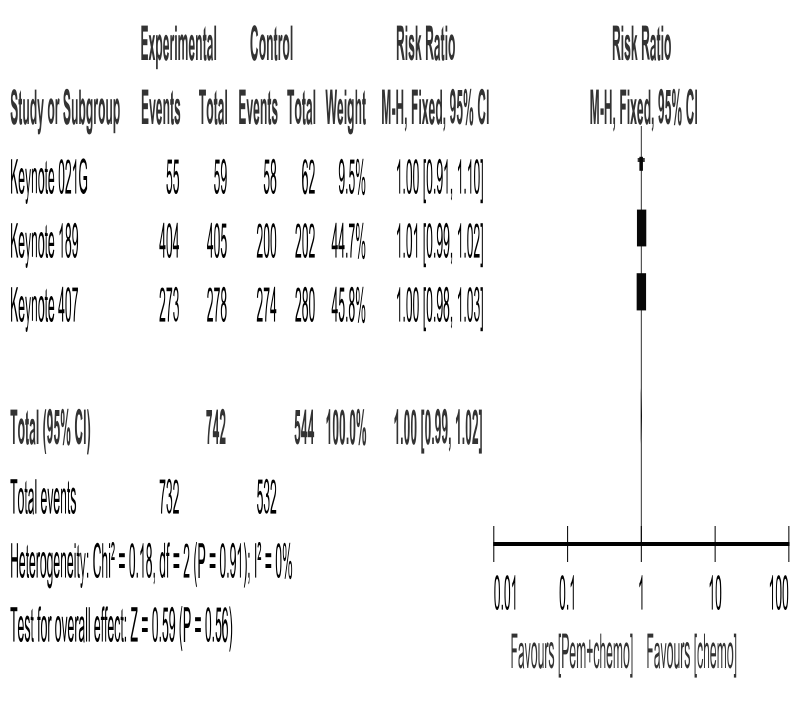


A


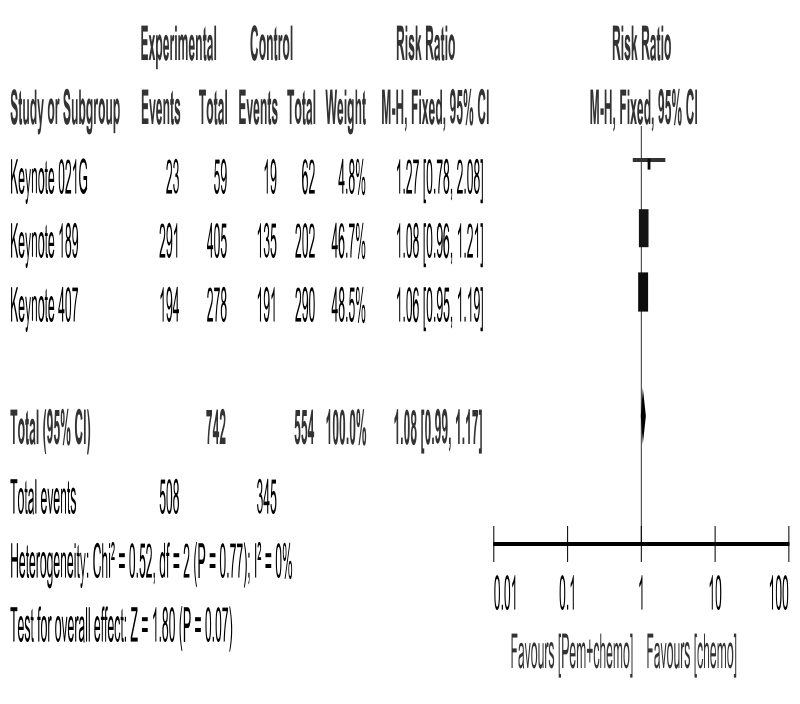

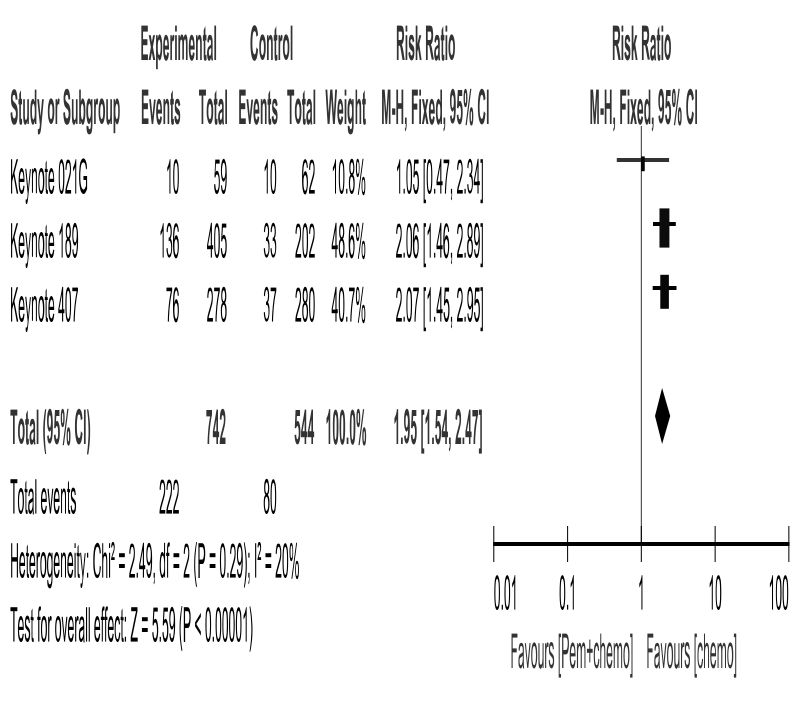

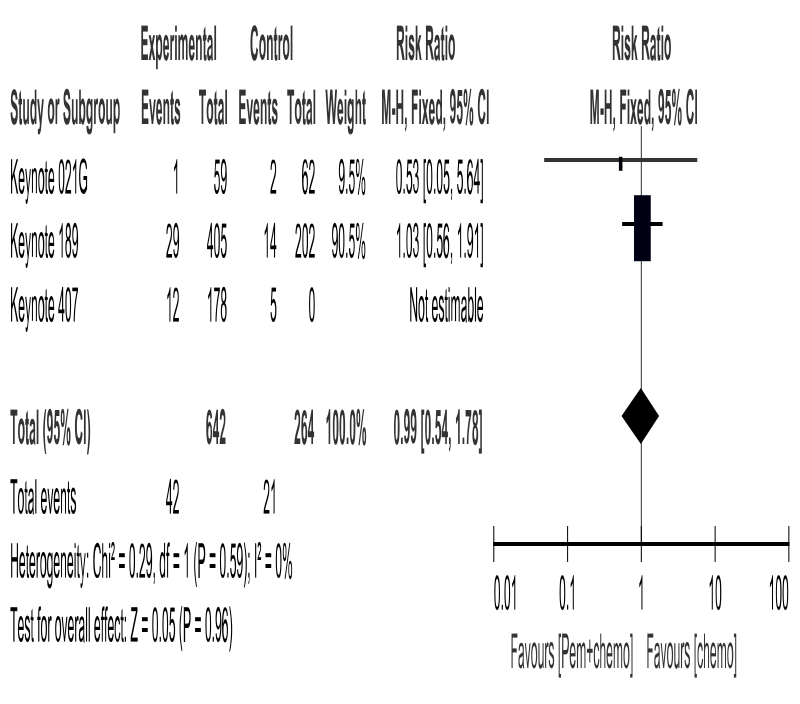


C

D

B
